# Widening Blockchain Technology toward Access Control for Service Provisioning in Cellular Networks

**DOI:** 10.3390/s23094224

**Published:** 2023-04-23

**Authors:** Fariba Ghaffari, Nischal Aryal, Emmanuel Bertin, Noel Crespi, Joaquin Garcia-Alfaro

**Affiliations:** 1 Samovar, Télécom SudParis, Institut Polytechnique de Paris, 91120 Palaiseau, France; 2 Institute of Research and Technology b-com, 35510 Cesson-Sévigné, France; 3 Orange Innovation, 14000 Caen, France

**Keywords:** blockchain technology, distributed ledger, smart contract, access control, development, private cellular network

## Abstract

The attention on blockchain technology (BCT) to create new forms of relational reliance has seen an explosion of new applications and initiatives, to assure decentralized security and trust. Its potential as a game-changing technology relates to how data gets distributed and replicated over several organizations and countries. This paper provides an introduction to BCT, as well as a review of its technological aspects. A concrete application of outsource access control and pricing procedures in cellular networks, based on a decentralized access control-as-a-service solution for private cellular networks, is also presented. The application can be used by service and content providers, to provide new business models. The proposed method removes the single point of failure from conventional centralized access control systems, increasing scalability while decreasing operational complexity, regarding access control and pricing procedures. Design and implementation details of the new method in a real-world scenario using a private cellular network and a BCT system that enables smart contracts are also provided.

## 1. Introduction

Blockchain technology (BCT), as the foundational design of distributed ledger technology (DLT), has revolutionized many aspects of business models and operations. Based on the traditional idea of hash chains to assure the integrity of data over distributed computing scenarios, BCT is believed to bring innovative new solutions, due to its key characteristics, such as decentralization, transparency, and immutability. Decentralization relies on the distribution of nodes over a global network, whose records are stored in registers (e.g., blocks) containing every transaction initiated in the system. All transactions are verified by multiple entities and securely recorded several times, through the use of encryption keys and electronic signatures. Records cannot be reversed, modified, or repudiated, thus creating an irrevocable and verifiable history of transactions. Registry management is decentralized and operates without a control body or centralized storage.

The use of BCT has received increasing attention, with Bitcoin [1] being the most famous application in the realm of cryptocurrencies, as well as Ethereum [2] and the extension of the concept of smart contracts [3], to autonomously execute agreements reached between distributed nodes. Together, these methods offer new possibilities to validate data transactions while offering traceability in a wide range of complex scenarios, far beyond the original usage of cryptocurrencies [4]. Examples include copyright management, sharing healthcare information, supply data, and real state control [5,6,7]. The intrinsic advantages of BCT are expected to change many aspects of business models, management, and operations in a range of fields. The list of major actors who reportedly explore BCT grows almost weekly [8]. Their expectations may vary and include potential cost savings, maintenance of technology leadership, and securing future business models.

In this paper, we focus on the establishment of trust in service provisioning ecosystems via BCT. The three main actors in need of the establishment of trust are users, mobile network operators, and service providers. For service provisioning through a mobile network, the conventional subscription and access management procedures are as follows. First, users need to subscribe to a given Mobile Network Operator (MNO) (which generally is a prepaid service) for calls and internet connections. In parallel, users also need to subscribe to the services provided by other providers (e.g., for video streaming, calls, storage services, and online games). Note that the services are often prepaid, i.e., users need to pay a fixed amount of money regardless of using the services in the end. In the access control and payment steps, the Service Provider (SP) needs to authenticate and authorize the user based on the information stored in the centralized server. While using the service, users shall pay both MNO (by decreasing its remained internet capacity) and SP (via prepaid or per-service pricing models).

The aforementioned scenario suffers from several drawbacks. First of all, the use of a centralized architecture for access control, by SP, leads to single points of failure [9] and low scalability. Moreover, centralized architectures impose higher complexity in IT operation, management, and maintenance. Since access management in conventional systems is handled in centralized servers or centralized parties, it can not only increase the processing load and overhead at the central point, but also reduces Quality of Service (QoS) [10]. Finally, it leads to high maintenance costs, since the maintenance of the central system and the provision of services to a large set of potential users is an expensive and complex endeavor. We propose to address the aforementioned challenges using a BCT-based solution for service provisioning in cellular networks. Our contributions include the outsourcing of an access control-distributed solution via a smart contract that decreases operation load in SP and MNO, while eliminating the need for trusted third parties. It also provides the automation of both access control and pricing procedures in service provisioning for SP and MNO, introducing new pricing models based on prepaid and Pay-As-You-Go (PAYG) scenarios.

The remaining sections are organized as follows. Section 2 provides the background on BCT, smart contracts, and access control methods. Section 3 presents our concrete BCT application, based on a decentralized access control as a service via smart contracts, to illustrate a representative new business model using BCT ideas. Section 4 provides our results. Section 5 discusses the advantages and limitations of our solution. Section 6 provides related work. Section 7 concludes the paper.

## 2. Background

In this section, we provide a comprehensive introduction of the essential preliminaries for the rest of the paper. First, we discuss Blockchain and distributed ledger technologies, as well as their operation workflows, benefits, and features. Then, the concept of smart contracts and their operation steps, as the most common extension of this technology, are presented. Finally, concerning our proposed system (i.e., a distributed access control solution relying on BCT benefiting from the smart contracts), some representative access control solutions and their workflows are described in the last subsection.

### 2.1. Blockchain and Distributed Ledger Technologies

Blockchain technology’s (BCT) proposal dates from 2009 [1], and it is tied to the Bitcoin cryptocurrency, where a practical implementation of the traditional concept of hash chains to ensure data integrity was revisited. A hash chain is the successive application of a cryptographic hash function to continuous flows of data transactions. In BCT, the authenticity of such transactions is also assured by digitally signing them by a sender, before broadcasting the result to a peer-to-peer network [11]. Special BCT users in that peer-to-peer network, known as miners. The term *miner* is commonly used in consensus models derived from the Bitcoin cryptocurrency. For the other consensus models, terminologies such as *block validator* or *leader* can replace the term *miner*). In the realm of cryptocurrencies, miners perform two main validations on the transactions. First, miners validate the correctness of the digital signature associated with the transaction. Second, miners validate that the the transaction is logically valid based on the policies in the network (e.g., in the case of a cryptocurrency, miners validate that the traded asset belongs to the sender and does not involve any double spending). If both validations succeed, the miners include the transaction in the next block of the chain, hence validating the consensus protocol underlying the network governance.

**Figure 1:**
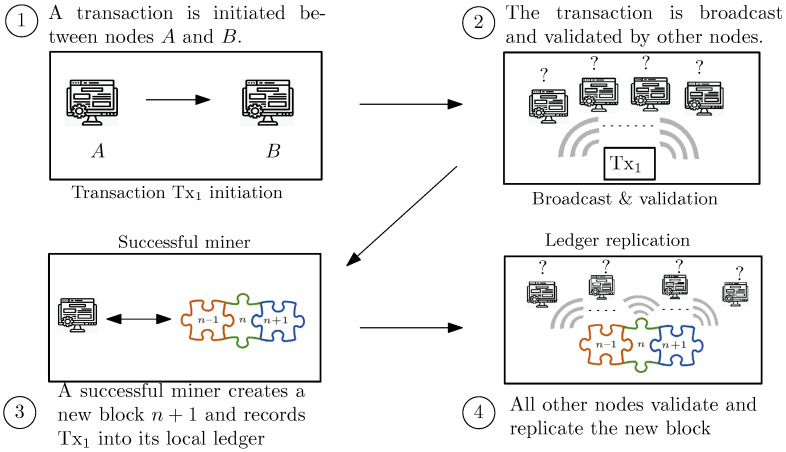
Use of a BCT system for trading assets between two nodes *A* and *B*. The ‘?’ symbol in Steps 2 and 4 refer to the execution of a given procedure by network nodes (e.g., transaction validation process in Step 2, and block replication in Step 4).

The workflow of processing the transactions and block generation in the BCT is depicted in Figure 1, in a simple example of trading between two entities. First, a transaction is initiated and broadcast to other nodes in the network. Then, the nodes which receive the transaction use the digital signature to verify the authenticity of the transaction. Once a transaction is validated, it is included in the list of valid transactions in the nodes. In the third step, the miners need to record the transaction into a new block and add it to the chain. To record the verified transactions, miners work to publish the new block (e.g., in the consensus model associated with the Bitcoin cryptocurrency, miners conduct this procedure by finding a potential Nonce to reach an agreement) and store a block of transactions in the ledger. Finally, other miners can then verify all the transactions stored in the block via the Merkle root. If all other verification steps hold correct information (e.g., authentication, integrity, lack of double spending, etc.) the new block is also added to their local replicas of the ledger. Indeed, two main concerns in this simple example (as well as other Blockchain-based distributed systems) are the lack of centralized authority to manage the synchronization of the transactions, as well as their ordering, and the integrity of data.

Addressing the challenges of data transaction ordering, synchronization, and integrity of data in BCT is done by the difficulty of rolling back in the chain of blocks. This provides strong immutability in BCT that makes the partial chain rendering unfeasible. In other words, by hashing the preceding block and inserting this amount as a header in the current block, any simple modification of a transaction included in previous blocks would require solving a consensus problem for all of the subsequent blocks. To assure that the whole chain of blocks becomes immutable, the inclusion of a new block in the BCT system must entail a given degree of difficulty. This is conducted by requesting the miners to perform some particular tasks defined in the consensus algorithm that may differ from every specific BCT implementation.

In addition to immutability, many other opportunities are provided by BCT designs. First of all, BCT allows for the creation of a new distributed paradigm, in which not only there is no centralized authority to control the network, but network failures are handled in a distributed manner. As a result, BCT-based systems can provide a high level of availability and fault tolerance. The idea to achieve this is the following. All nodes in a BCT system rely on consensus theory (i.e., a well-established sets of rules and algorithms to ensure that everyone behaves as expected). This assures the integrity-by-design feature. Additional properties associated with BCT systems include *traceability, transparency, non-repudiation* and *permanence*. Traceability and transparency mean that all data transactions are available to be seen and tracked by any nodes with access to the system. In other words, data must always be available and traceable at any time. Non-repudiation in BCT means that nobody can deny their actions in the system. The use of cryptography in BCT ensures that all parties must digitally sign their actions, hence avoiding the possibility of action denial by BCT entities. Finally, permanence means that all data in a BCT can be available at any time (nothing may be removed from the network).

**Figure 2:**
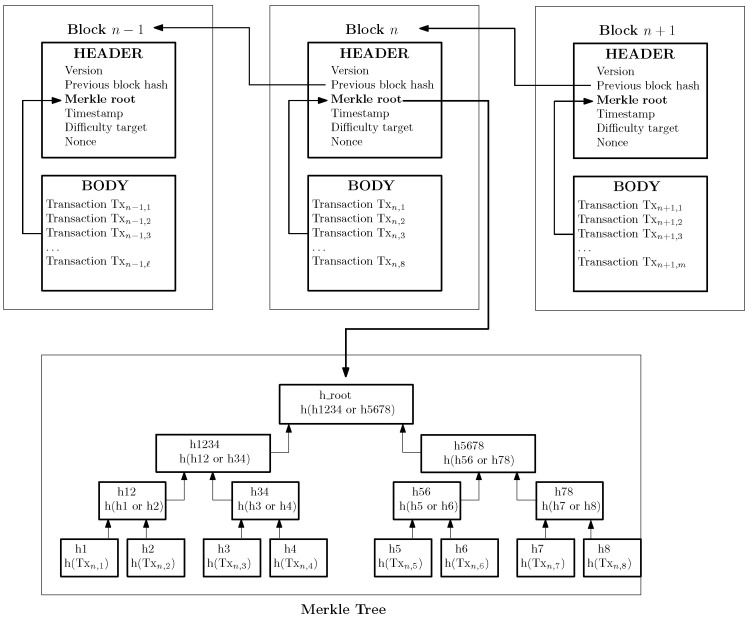
Structure of a BCT design and its use of Merkle trees.

In terms of data structures associated with BCT, blocks are composed of a body and a header (cf. Figure 2). The body stores the transaction data. The header contains metadata, such as the hash of the previous block, a timestamp, a cryptographic Nonce (i.e., a number used only once, for security purposes), and a Merkle root. The hash value is calculated by passing the header of the previous block to a hash function. The timestamp is used to keep track of the specific creation time of the block. The Merkle tree is a binary data structure, in which each leaf node is labeled with the hash of one transaction stored in the block body, and the non-leaf nodes are labeled with the concatenation of the hash of its child nodes. The Merkle root represents the root hash of the Merkle tree. It is used for performance purposes, such as optimizing the search time during the verification of transactions contained in the Blockchain. Any modification affecting a transaction (i.e., even a bit-flip) will render to a different Merkle root; hence verification and comparisons can be conducted by simply looking at the Merkle root of the block, without the need to go through all the transactions stored in a block.

The more general concept of distributed ledger technology (DLT) includes aforementioned designs around the concept of blocks and Merkle trees, as well as alternative designs, including different data structures, consensus algorithms, and governance solutions. Blocks can be replaced by other non-linear data families, including with the use of directed acyclic graph (DAG) or any other hybrid data structures. Different designs can be classified depending on the rule that regulates as to which nodes can access, verify and validate the transactions in the system [12]. DLT platforms associated with Bitcoin and Ethereum represent the idea of permissionless *(public)* ledgers, i.e., DLT designs whose ledgers are accessible to the public. In other words, these are designs in which any participant can broadcast new transactions, participate in the consensus procedures, write into the ledger, etc. While Bitcoin and Ethereum mainly represent existing DLT examples underlying contractual decentralized transaction models, some other existing frameworks extend them to address applications in different domains, such as https://ripple.com/ (accessed on 21 February 2023), for banking applications, https://www.energyweb.org/ (accessed on 21 February 2023), for energy, https://www.hyperledger.org/ (accessed on 22 April 2023), for supply chain, logistics, and much more, etc. In all those previous cases, and by granting the authority of maintaining a ledger to all the nodes, public ledgers may also become fully distributed and even allow scenarios with anonymity requirements. However, these systems suffer from the low speed of transaction validation and require a certain level of computation to secure them regarding the intrinsic vulnerabilities of DLT. Next, we provide some additional information on existing DLT-models.

Permissioned designs can be used to construct either *private* or *consortium* DLT-platforms. The former is related to the solutions that are usually maintained by a single organization, i.e., the ledger is developed in specific organizations based on their needs. The rights to access the ledger and to verify the transactions are granted through a central controller to the permissioned nodes. A permissioned network is thus established, in which only the authorized nodes can access certain transactions of the ledger or participate in working to publish new blocks. Due to having a minimum level of trust among the nodes in the permissioned network, the computation-intensive consensus algorithm can be either omitted or replaced by a simpler algorithm. Hence, the secrecy of the transactions is highly improved and the decentralization of authority of transaction validation is under the control of the organization. A second subcategory of permissioned designs leads to *consortium* ledgers, which are similar to the private ones, in the sense that they are maintained in a permissioned network, but differ from private ledgers, since they involve multiple organizations to share the right to access and validate the transactions. Although these organizations might not fully trust each other, they can work together by altering the consensus algorithm, based on the level of trust among them. Consortium platforms can also be used as a distributed and reliable database for predefined enterprises for business-to-business purposes. However, only the eligible nodes, defined by participating organizations, can join in the consensus process. The anonymity of users can be violated. Moreover, the use of tokens or fees is not mandatory for the process or for validation of transactions.

For the sake of simplicity, we assumed in previous explanations that to reach transaction validation, it suffices to validate the correctness of digital signatures associated with the transactions, as well as asset ownership and double spending avoidance. However, this minimal set of conditions can be extended to reach some more complex agreements. This can be achieved using smart contracts. In the following, we provide some additional information about it.

### 2.2. Smart Contracts Enabled by BCT

Originally defined in the mid-1990s [3], the concept of smart contracts refers to automated agreements among mutually distrusting parties [13], without the need for a trusted intermediary. Users can request the execution of a smart contract via peer-to-peer network transactions of a distributed ledger, i.e., each execution request gets logged into a public, append-only Blockchain. Potential conflicts in the execution of each execution requests are also handled through the distributed ledger, i.e., using its associated consensus protocol.

The operation of a smart contract is briefly described next, with a representative example [14], in which an entity *A* agrees to remunerate a second entity *B* for setting up a new service. The remuneration is expected to be conducted in two steps. First, 70% of the total remuneration is assumed upon completion of an initial configuration step. Then, 30% of the total remuneration occurs two months after the full completion of the service, thus making it possible to validate the service of *B*. The contractual establishment is conducted in two main stages. During the first stage, entities *A* and *B* establish a smart contract between them. *A* and *B* have assumed valid accounts related to a given Blockchain. The smart contract between *A* and *B* is also stored over the Blockchain, as a special entity that conceptually resembles the instantiation of an object and which defines various operational rules in the form of publicly exposed methods. Such methods determine a change in the state of the instantiated object, i.e., the state of the smart contract. Once the conditions of the smart contract are validated by the corresponding parties (e.g., miners, validators, etc.), we assume that the contract starts its execution. With respect to traditional execution environments, the immutable nature of BCT is materialized in an execution that is virtually impossible to be stopped (i.e., once the execution of the contract is launched, it cannot be reverted).

Notice that the previously formalized agreement between *A* and *B* is executed upon the ledger in an automated manner. Each change of state of the program is a recorded transaction. Neither of the two parties (nor anyone else) can perturb the execution of the contract. The stages of the service associated with *B* are checked automatically, and if these are carried out, the planned contributions are rewarded to *B* in the form of transactions associated with *B* within the ledger itself. In the end, whenever all the conditions (i.e., agreements) are met, the transaction is completed and *B* becomes the owner of the assets. Otherwise, if the conditions are not properly satisfied, *A* remains the owner (i.e., *A* recovers the amount due). The deployment of smart contracts on top of a DLT platform, such as Ethereum, can generally be done by the development of decentralized application (DApp), which are digital applications or programs that exist and run on a distributed network of computers instead of a single computer (cf. Section 3 for further details about more elaborated smart contract examples). In terms of data structures, platforms such as Ethereum also generalize the approach depicted in Figure 2, with some more elaborated data structures. Each block header (w.r.t. the approach depicted in Figure 2) can now be tied to several trees; one tree is used to store the transactions, another is used to store the state, and a third tree is used to store the results. The combination of Merkle trees together with Patricia trees [15] is the fundamental data structure on which Ethereum is built. However, a more elaborate presentation of these structures is left out of the scope of this paper.

Before moving forward, and presenting our proposal concerning a concrete application over a specific BCT system enabling smart contracts, we provide next some necessary background on representative access control solutions and preliminaries of the private cellular networks in the following two subsections.

### 2.3. Access Control Models

Access control regulates who or what (i.e., a subject) can perform which action (or have which permissions) upon an object (e.g., network resource, applications, services, databases, etc.) [16]. The access control procedure is done in three main steps. We must first define a series of rules associated with a policy, determining the conditions for accessing an object. Each rule definition is varied based on the access control model. Secondly, access verification is conducted, in which the access control server examines the received access request based on a subject’s permissions. If they match, an access solution based on the enforcement method will be assigned to the subject. The recording of access logs completes the third step, in which all activities of the subjects and their accesses will be recorded.

Several well-known access control methods are introduced as follows. The Discretionary Access Control (DAC) model [17] considers the owner-based administration of objects. More precisely, the owner of an object defines the access rules and policies.DAC can be implemented via an Access Control List (ACL) that defines as to which objects can be accessed by what subject and with what type of permission. A similar access control method is Capability-based Access Control (CapBAC) [18], in which a capability is associated with each subject and used for access management. In CapBAC, users are granted access permissions based on an access token, such as a key, a ticket, or a credential. When a system aims to manage a large number of assets, CapBAC and DAC decrease the manageability [19]. Hence, Role-Based Access Control (RBAC) is developed to resolve this challenge. It manages the subjects’ access, based on their role within the system, and defines what kind of accesses are associated with the subject of a given role [20].

Attribute-Based Access Control (ABAC) [21] is a logical model that controls the access to objects by evaluating some defined access control rules or policies in terms of the *subject, object, action* and *environment* attributes. Fine-grained access management of ABAC makes this solution a primitive candidate for our proposed method. ABAC is fine-grained because it supports different constraints in order to define the legitimate user. Furthermore, it provides dynamic and context-specific access, which makes the resource owner capable of defining the access control policy based on their needs. ABAC generally uses Boolean logic, in which the policy controller can verify the subject’s eligibility in Boolean logic, based on four sets of attributes to define the access policy and manage the subject’s access to the object. These sets are Subject, Object, Environment, and Action attributes.

Subject attributes specify the subject by its identifiers, such as its username, token, and so on. Object attributes distinguish the resources that the subject wants to access, for instance, the file name, the network resource, the service name, etc. Action attributes are the actions that can be performed by the subject (e.g., *read*, *write*, and *execute*). Finally, Environment attributes describe the context in which access is requested (e.g., the time and location of the access request, or the type of communication channel). After receiving the object’s request, policy controllers would validate this request based on the defined rules in terms of these four attributes, and the access control result is returned as *allow* or *deny* to the user.

Once establishing the building blocks in the area of access control solutions, we present, in the following subsection, some needed preliminaries on private cellular networks, in order to establish the use of the concrete application promoted in this paper.

### 2.4. Private Cellular Networks

Private Cellular Networks (PCNs) are local area networks that leverage radio access technologies (e.g., 4G Long Term Evolution (LTE) and 5G technologies) to establish a dedicated network and meet the requirements specific to an organization (e.g., schools, industrial sites, and smart cities). PCNs use small cell towers to cover a defined location, and the organization is responsible for the core network management. By using Subscriber Identity Module (SIM) cards exclusive to the network, only selected devices can connect to it.

PCNs can be considered to be small-scale versions of public cellular networks. The main difference between private and public networks is the control over the overall aspect of the network [22]. The infrastructures and resources in public networks are shared, whereas private networks control all network infrastructures and resources. At the same time, PCNs are suitable for services that require high availability and performance [23]. Other advantages of private networks include lower cost, better connectivity, improved security, higher scalability, and good coverage [24,25]. These factors have led to a great interest in private networks from both business and academia.

It is crucial to have a lab deployment before private cellular networks are used in real life to test and validate any theories [26]. There are many open-source programs available to build private cellular networks. For our purposes, we utilize OpenAirInterface (OAI) and Magma core, because they are the two primary open-source projects that allow the flexible deployment of small-scale mobile communication systems, but with two contrasting ideas in their architecture and execution.

OAI is an open-source platform to provide services for the software-based implementation of different cellular network infrastructures, such as user equipment, radio access networks, and core networks [27]. OAI complies with standards from the 3GPP partnership program. It supports 4G LTE and 5G broadband cellular network implementations. Along with the software version, OAI provides features to integrate commercially available hardware, such as smartphones, programmable SIM cards, and universal software radios, for transceiver functionality. On the other hand, the Magma core is an open-source implementation of the cellular core that supports 4G LTE and 5G radio access technologies. It consists of three major components: the Access Gateway, the Orchestrator, and the Federation Gateway. The Access Gateway (AGW) represents the core network services and policies. It uses the evolved packet core for LTE networks and the 5G core for 5G networks. It can be integrated with commercial radio hardware to provide network services. The Orchestrator is the cloud service responsible for configuring and monitoring network activities hosted on a public/private cloud. It provides a web platform for network configuration and a visual representation of network traffic flows. The Federation Gateway connects the MNO core to the Magma network via a standard interface (e.g., 3GPP interfaces). It acts as a proxy between the MNO’s network and the Magma AGW. Additionally, it handles the core functions (e.g., authentication and policy enforcement).

Compared to a typical mobile core implementation, Magma core does not support 3GPP interfaces between mobile core components [28]. However, it provides federation gateway interfaces and Radio Access Network (RAN) support for 3GPP protocols. Next, we will use OAI and Magma core to establish our BCT-based application, in order to to outsource access control and pricing procedures of cellular networks via smart contracts.

## 3. BCT-Based Access Control for Service Provisioning in Cellular Networks

We introduce in this section our BCT-based ABAC system for service provisioning in cellular networks, which provides the opportunity of introducing new business and pricing models in this market. In the current service-provisioning model using the mobile data in cellular networks, first, the user subscribes to a given MNO, in parallel to the services provided by other service providers. To access the services (as well as in the pricing step), SP authenticates and authorizes the user in their centralized servers. In this scenario, not only the user is subscribed to SP and is paying for that, but using the service consumes their mobile data in the network.

We have identified several drawbacks in this scenario. First, the central authorization server can be a single point of failure and limits the system’s scalability. Moreover, the processing loads and the complexity of IT operations in both SP and MNO are very high. Furthermore, several intrinsic issues of centralized access control solutions can defect these systems as well. For instance, these include the risk of losing the user’s data in a centralized server, denial of an action done by a malicious user because of low non-repudiation, the low immutability of rules in the system, and the high maintenance costs of the centralized server.

To address these challenges, our proposed ABAC model outsources the authorization and pricing procedures of service providers and MNOs to a distributed network containing a consortium of participating organizations. Moreover, this system proposes a new business model that can attract users by eliminating their payment to MNO while using the services provided through the platform (i.e., the user’s mobile data will not be used while using the service, and instead of that, either the service provider pays MNO on behalf of the user, or the user pays both the MNO and SP separately without using the mobile data).

The overall steps of access control and payment procedures of the proposed method are enumerated as follows:

1. The user registers in the provided DApp by sending a request to a dedicated smart contract through a transaction in the Blockchain.
2. The registration smart contract deploys a unique smart contract for the user (only for the first time).
3. User chooses their desired service from the list of available services for registration.
4. To access the service, the user sends an access transaction to the dedicated smart contract in the system.
5. The access manager smart contract authorizes the user regarding the stored policies of the requested service.
6. According to the pricing model of the service, the access manager smart contract blocks an amount of money.
7. After termination of the service usage, the access manager smart contract pays the MNO, SP, and (if it is required) the user, according to the pricing model.

Next, we detail the system architecture and the design of smart contracts.

### 3.1. System Design

**Figure 3:**
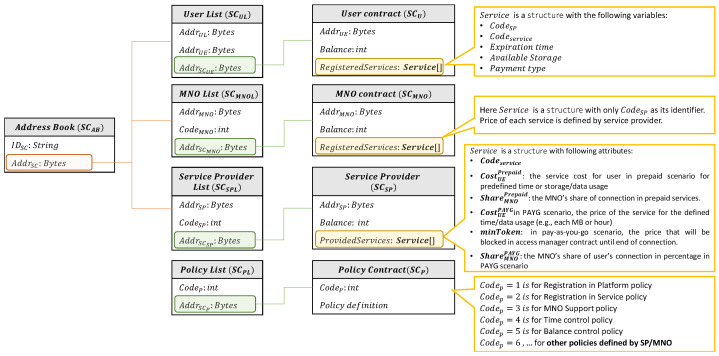
Relations and connections among designed contracts (excluding manager contracts).

In this section, we provide a brief description of the smart contract models, which handle the registration, access control, and payment procedures in the system. In this regard, we categorized the smart contracts into the following groups: (i) reference contracts; (ii) database contracts; (iii) policy definition contracts; (iv) manager contracts. Figure 3 depicts the connection between contracts and contract attributes, and their definitions.

Our two main general assumptions for deploying the proposed system are the following. First, all the connections between the user, the decentralized application (DApp), the service provider, and the MNO are secure. Second, an unlimited number of MNOs, SPs, and users can register to the system. More information about the precise contracts and the registration steps is presented in the following.

#### 3.1.1. Reference Contract

In this category, the *Address Book contract ($SC_{AB}$)* is defined to store a mapping of an identifier to the address of single smart contacts (i.e., $SC_{ReM}$, $SC_{ACM}$, $SC_{UL}$, $SC_{PL}$, $SC_{SPL}$, $SC_{NPL}$) as:

$$Entry_{AB}\overset{ID_{SC}}{\leftarrow }Addr_{SC}$$

where $ID_{SC}$ is a predefined unique identifier for smart contracts and $Addr_{SC}$ is its address in Blockchain. The primary objectives of designing this contract are as follows: (i) avoiding the use of hard-coded addresses to prevent potential maintainability issues of smart contracts [29], (ii) managing a list of addresses and implementing modifiers in functions to leverage the intrinsic access control capability of smart contracts, and (iii) facilitating secure collaboration among multiple parties involved in the contract.

#### 3.1.2. Database Contracts

Seven additional contracts are defined under this category:

- The *User List contract ($SC_{UL}$)*, *MNO List contract ($SC_{MNOL}$)*, and *Service Provider List contract ($SC_{SPL}$)* store the list of registered users, network providers, and service providers, mapped with the following structures, respectively:
  $$Entry_{UL}\overset{Addr_{U}}{\leftarrow }Addr_{SC_{U}}$$
  $$Entry_{MNOL}\overset{Code_{MNO}}{\leftarrow }Addr_{MNO}\overset{Addr_{MNO}}{\leftarrow }Addr_{SC_{MNO}}$$
  $$Entry_{SPL}\overset{Code_{SP}}{\leftarrow }Addr_{SP}\overset{Addr_{SP}}{\leftarrow }Addr_{SC_{SP}}$$
- *User contract ($SC_{U}$)*, which is a unique smart contract for a particular user which stores, at least, the user’s balance and all their registered services in the following structure:
  $$Attr_{U}\overset{Addr_{U},Code_{SP}}{\leftarrow }\left(Balance,Services\left[\right]\right)\overset{Code_{Service}}{\leftarrow }ExpTime,AvailableStorage$$
  where Balance is the user’s current balance in their wallet, Services[] is a list of the user’s application-layer services or specific subscribed services, ExpTime is the expiration time of the user’s access to that specific service (if it is not applicable, it can be set to 0), and the user’s available storage if the service is related to storage.
- *MNO contract ($SC_{MNO}$)*, which is a unique smart contract for a particular MNO, which stores, at least, their balance and all the services (or service providers) that the related MNO supports.
- *Service Provider contract ($SC_{SP}$)*, which is a unique smart contract for a particular service provider, which stores, at least, their balance in the system, all their provided services, and the list of applicable policies for each service.
- *Policy List contract ($SC_{PL}$)*, which stores the address of the policy smart contract, with a mapping to their code, as shown in
  $$Entry_{PL}\overset{Code_{P}}{\leftarrow }Addr_{SC_{P}}$$
  where $Code_{P}$ is the policy’s pre-defined code in the system, and $Addr_{SC_{P}}$ is the related smart contract that defines that policy.

#### 3.1.3. Manager Contracts

In this category, the following two contracts are introduced:

- A *Registration Manager contract ($SC_{ReM}$)*, which manages the user, network provider, and service provider registration procedure in the platform. The main functions of this smart contract are:
  – registerServiceProvider() that registers new service providers in the platform.
  – registerNewUser() that registers a new user in the system and deploys the user’s smart contract for the first time.
  – registerNewMNO() that registers new network providers in the platform. This registration needs the consensus of all other registered network providers.
  – registerToServicePrepaid() that registers the user in a prepaid service.
  – registerToServicePAYG() that registers the user in a PAYG service.
- *Access Control Manager contract ($SC_{ACM}$)*, which manages the user’s access control by validating the request against applicable policies for the requested service. Moreover, after the termination of the service usage, this contract handles the payment procedure. The main functions of this smart contract are:
  – userValidation() to validate the user’s eligibility to access the service regarding different policies and payment methods. Moreover, it blocks a specific amount of money inside the contract, as a distributed trusted party for the user, SP and the MNO, using the user’s address as the indicator, to manage the payment procedure after termination.
  – terminationAndPayment() manages the payment to the SP, the user, and the MNO.

#### 3.1.4. Policy Definition Contracts

For the first setup of the system setup, we define five rules to validate the request’s access attributes. Each policy is defined in the different smart contracts, and the service providers can add the list of applicable policies to their services. The following rules are set in the first setup of the system. If any SP needs to add a policy, such an SP deploys a smart contract for that and adds its code to their service.

- *Subject attribute validation*, which includes a series of policies to validate the user’s registration, as follows:
  – *Registration in Platform contract ($SC_{RP}$)*: validates if the user is registered in the platform.
  – *Registration in Service contract ($SC_{RS}$)*: validates if the user is registered in the specific requested service of the particular service provider.
- *Environment attribute validation*, which includes another series of policies to validate if the user is eligible to use the service, as follows:
  – *MNO Support contract ($SC_{MNOS}$)*: validates if the user’s MNO supports the user’s demanded service.
  – *Time Control contract ($SC_{TC}$)*: validates if the service’s expiration time is not passed for that specific user.
  – *Balance Control contract ($SC_{BC}$)*: validates if the user has enough balance to register and access the service in the following scenarios:
    – For the prepaid services, the user’s balance needs to be checked in the registration step.
    – For the prepaid services, the service provider’s balance needs to be checked in the access control step.
    – For the *PAYG* services, the user’s balance needs to be checked in the access control step.

### 3.2. Registration Step

In this step, the registration procedure of SPs, MNOs, and users is described.

#### 3.2.1. Service Provider Registration

**Figure 4:**
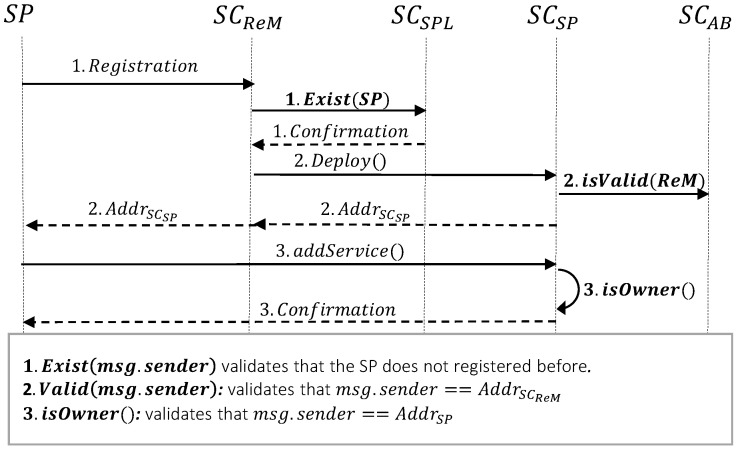
Service provider registration steps.

The registration of the SP in the system is completed via the following steps (cf.  Figure 4 for a more detailed description of this procedure):

1. First, SP sends the registration request to $SC_{ReM}$. Since each service provider can register only one time, $SC_{ReM}$ needs to verify that the SP is not registered beforehand. To do so, it calls the isExist() function of $SC_{SPL}$ and sends the address of the caller as its argument. Note that here the caller is SP, so $SC_{SPL}$ sends the $Addr_{sp}$ in isExist() function. Since msg.sender in Solidity language is the address of the caller or the creator of the transaction, in the rest of the paper we use msg.sender to indicate the caller of the function.
2. After receiving confirmation from $SC_{SPL}$, $SC_{ReM}$ deploys the service provider’s unique smart contract (i.e., $SC_{SP}$). Note that the deployment of smart contracts for all entities in the system is only can be done by $SC_{ReM}$. Therefore, the constructor() of $SC_{SP}$ verifies that msg.sender is equal to $Addr_{SC_{ReM}}$. It is important to mention that in the proposed system, the only fixed and hard-coded address is $Addr_{SC_{AB}}$, to be able to use this smart contract as a reference point. After the deployment of $SC_{SP}$, its address is sent to SP.
3. Finally, SP as the owner of the smart contract can add its preferred services into $SC_{SP}$. These services would be advertised to the network providers and the users for further registration. While inserting the services into $SC_{SP}$, the service provider defines the costs for prepaid and PAYG scenarios (note that the service providers can choose one of these payment solutions based on their preference). The following costs will be added to $SC_{SP}$ for each advertised service (cf. Figure 3, $SC_{SP}$):
  - $Cost_{UE}^{Prepaid}$: defines the prepaid cost that the user needs to pay for registering in this service for a predefined time/data usage.
  - $Share_{MNO}^{Prepaid}$: defines the fee that SP will pay to MNO on behalf of the user, after user access termination.
  - $Cost_{UE}^{PAYG}$: defines the fee that the user needs to pay per hour/MB while using a PAYG service.
  - minToken: defines the minimum required tokens in the user’s wallet to give access to a PAYG service. Note that the user’s real usage may be more than this amount, so, the user will be charged after access termination for the remaining part. or, if the real cost is less than this amount, the user’s wallet would be refunded.
  - $Share_{MNO}^{PAYG}$: defines the MNO’s share in percentage from the user’s real usage of service. So, the cost of the user’s real usage will be between SP and MNO based on this value.

#### 3.2.2. MNO Registration

**Figure 5:**
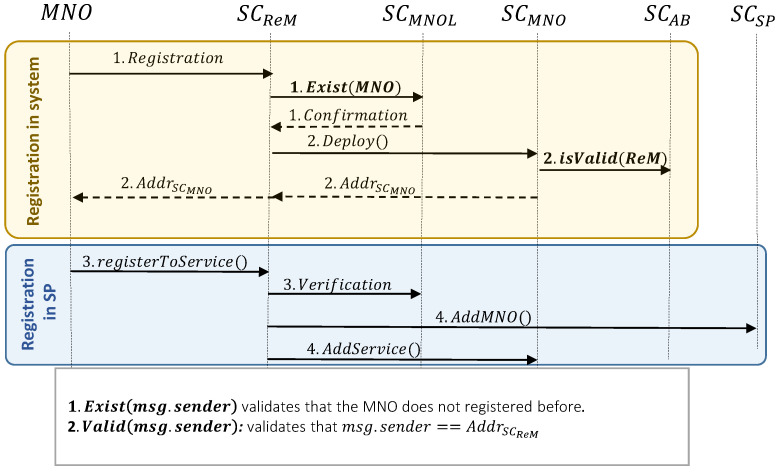
MNO registration steps for the platform and service provider.

The registration of MNOs consists of two main phases. First, they register to the system to insert their address in $SC_{MNOL}$, and then they register to the provided service by the service provider, to let their user use the service with the new proposed business model. At the same time, the MNO registration steps in the system and service provider are as follows (cf.  Figure 5 for the precise workflow):

- *MNO registration in the system:*
  1. MNO sends the registration request to $SC_{ReM}$. Similar to the service provider registration procedure, each MNO can register only one time to the system. So, $SC_{ReM}$ verifies that the MNO is not registered beforehand (i.e., by using the isExist() function of $SC_{MNOL}$ and sending the MNO address, msg.sender, as its argument).
  2. After receiving confirmation from $SC_{MNOL}$, $SC_{ReM}$ deploys the MNO’s unique smart contract (i.e., $SC_{MNO}$), after verifying that msg.sender $==Addr_{SC_{ReM}}$. After registration, MNO as the owner of the smart contract can add its preferred services into $SC_{MNO}$, using the following steps.
- *MNO registration in the services:*
  3. MNO selects the desired services from existing options, then sends the registration request to $SC_{ReM}$. After receiving the request, $SC_{ReM}$ verifies that MNO is already registered in the system. Note that, when MNO selects a service from existing services in the system, this means that it agrees on the prices that are related to the payment to MNO (i.e., $Share_{MNO}^{Prepaid}$ and $Share_{MNO}^{PAYG}$).
  4. To register the MNO is an specific service, $SC_{ReM}$ inserts the $Code_{MNO}$ and $Address_{MNO}$ into $SC_{SP}$. So, the service provider would have the MNO in its customer list. Moreover, the $Code_{SP}$ and $Code_{service}$ will be inserted in $SC_{MNO}$ as well.

#### 3.2.3. User Registration

**Figure 6:**
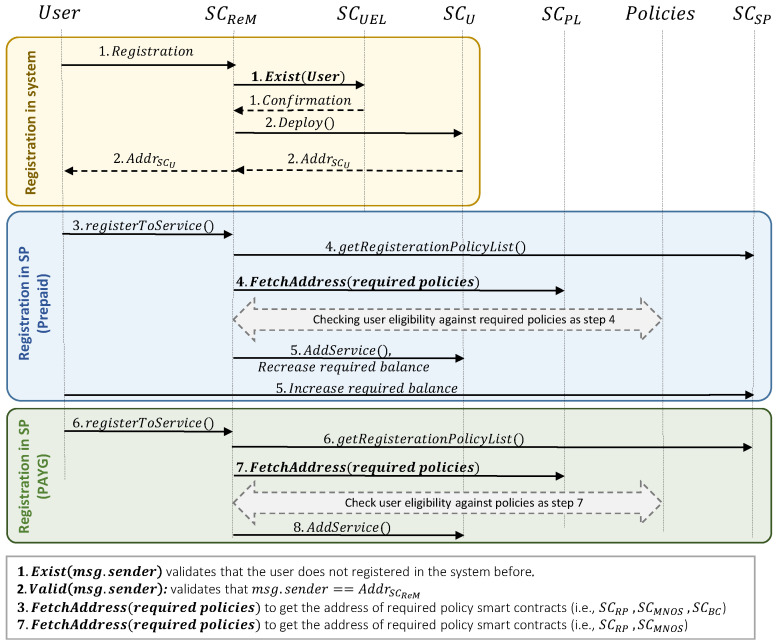
The user registration steps of the platform and service provider for prepaid and PAYG pricing methods.

Figure 6 depicts the procedure associated with the user registration in the system and service provider. It works as follows:

- *User registration in the system:*
  1. UE sends the registration request to $SC_{ReM}$. Since each user can register only once to the system, $SC_{ReM}$ verifies that the UE has not been registered beforehand (i.e., by calling the isExist() function associated with $SC_{UEL}$ and sending the value of $Addr_{UE}$ (msg.sender) as its argument).
  2. After receiving confirmation from $SC_{UEL}$, then $SC_{ReM}$ deploys the unique smart contract of the user (i.e., $SC_{UE}$).
- *User registration in the prepaid pricing model:*
  3. UE selects the desired services from existing options, then sends the registration request to $SC_{ReM}$. After receiving the request, $SC_{ReM}$ fetches all policies that are defined for the registration of the user in the system (e.g., checking the user’s balance and verifying that $Balance_{UE}\geq Cost_{UE}^{Prepaid}$).
  4. Once getting the list of policies, $SC_{ReM}$ will retrieve the address of each smart contract in which those policies are defined (e.g., $SC_{RP}$, $SC_{RS}$, $SC_{MNOS}$, $SC_{TC}$, $SC_{BC}$). Then it can verify the user’s eligibility based on each policy.
  5. In case of the user’s eligibility, the $Code_{SP}$ and $Code_{service}$ will be inserted into $SC_{UE}$. Moreover, the $Cost_{UE}^{Prepaid}$ will be transferred from the user to the service provider’s wallet. Note that this transfer is based on ERC20 standards [30].
- *User registration in the PAYG pricing model:* Because the majority of the registration steps in this model are the same as for the prepaid one, we only summarized the main steps.
  6. UE sends the registration request to $SC_{ReM}$, then it fetches all required registration policies.
  7. $SC_{ReM}$ verifies the user’s eligibility based on each policy.
  8. $Code_{SP}$ and $Code_{service}$ will be inserted into $SC_{UE}$. In this pricing model, the user does not need to make any payment in the registration step.

### 3.3. Attribute-Based Access Control

**Figure 7:**
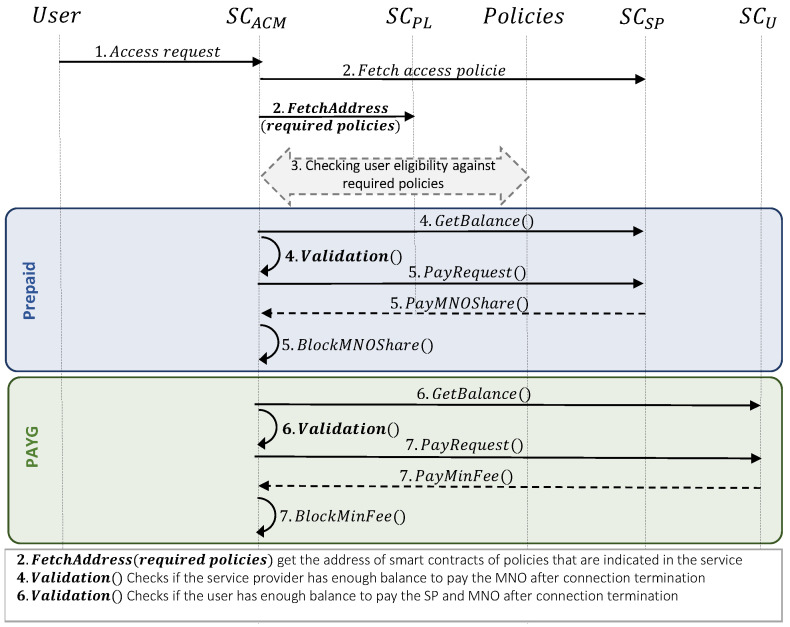
The ABAC procedure for user access to the services in the prepaid and PAYG scenarios.

After successful registration of the entities in the system and services, the users are able to access these services through the proposed system. The access control procedure for different pricing methods is enumerated as follows (see Figure 7):

- *User access verification:*
  1. UE selects a service among registered services, (this selection creates an access request transaction to $SC_{ACM}$ smart contract).
  2. After receiving the request, $SC_{ACM}$ fetches all policies that are defined as prerequisites for access o the service (e.g., checking the user’s balance, checking the geographical IP, etc.).
  3. After getting the list of policies, $SC_{ACM}$ retrieves the address of each smart contract in the list (e.g., $SC_{RP}$, $SC_{RS}$, $SC_{MNOS}$, $SC_{TC}$, $SC_{BC}$). Then it can verify the user’s eligibility based on each policy (i.e., for the verification we defined a isEligible() function, that compares the user’s access attributes with the defined rules).
- *User access control to the prepaid pricing model:*
  4. If the access verification is successful, $SC_{ACM}$ validates the service provider’s balance for further user access. It is important to mention that, in the prepaid pricing model, the user is paid to the service provider while the registration step, and while using the service, the user would not pay to MNO (e.g., the user’s mobile data will not be reduced while using the service); and, the service provider is the entity that will pay to MNO on behalf of the user. So, $SC_{ACM}$ verifies that $Balance_{SP}\geq Share_{MNO}^{Prepaid}$.
  5. If the balance verification is successful, the $Share_{MNO}^{Prepaid}$ will be transferred from the service provider’s wallet to $SC_{ACM}$ as a distributed trusted party for all entities. Note that, this transfer is based on ERC20 standard [30]. Record of this payment is added to $SC_{ACM}$ as a mapping of the user’s address to a balance as follows:
    $$Balance_{U}\overset{Addr_{U}}{\leftarrow }StoredBalance_{U}$$
- *User access control to the PAYG pricing model:*
  6. If the access verification is successful, $SC_{ACM}$ validates the user’s balance, since, in this pricing model, the user needs directly pay the service provider and MNO separately, according to the real service utilization. So, $SC_{ACM}$ verifies that $Balance_{UE}\geq minToken$.
  7. If the balance verification is successful, the minToken will be transferred from the user’s wallet to $SC_{ACM}$. Note that this amount balance is only a minimum balance to guarantee the payment to the service provider and MNO. It means that the user’s real utilization will be sent to $SC_{ACM}$ after termination, and the real price will be calculated at that time. Same as in the prepaid model, the record of this payment is added to $SC_{ACM}$.

### 3.4. Payment

**Figure 8:**
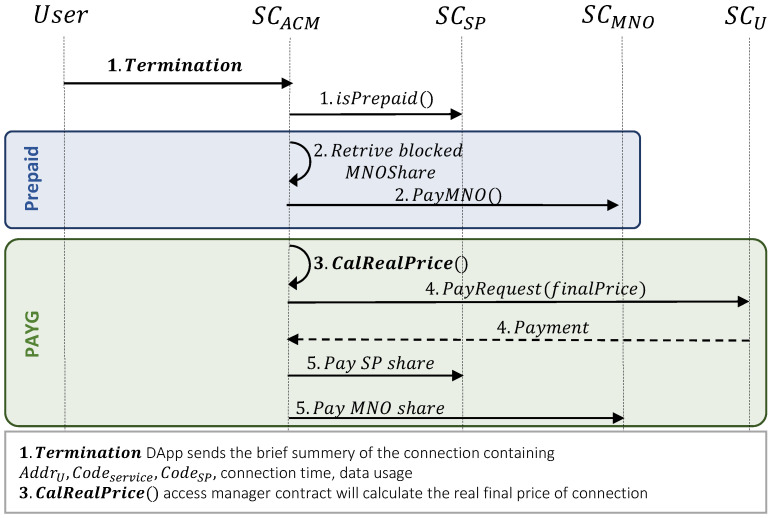
The payment procedure to service provider and MNO in the prepaid and PAYG scenarios.

Once the user terminates the service utilization, the pricing and payment procedure will be executed as follows:

- *Checking service type:*
  1. UE sends the termination transaction to the $SC_{ACM}$ smart contract. This contract checks the service type for handling the further payment procedure.
- *Payment in the prepaid pricing model:*
  2. In the prepaid pricing model, once $SC_{ACM}$ receives the termination transaction, it retrieves the blocked $Share_{MNO}^{Prepaid}$ and pays it to MNO (see Figure 8). This transfer complies with the ERC20 standard [30].
- *Payment in the PAYG pricing model:*
  3. First, $SC_{ACM}$ calculates the real service price as follows:
    $$FinalPrice=Usage\times Cost_{UE}^{PAYG}$$
    Then, it calculates the amount of money that the user needs to pay or be reimbursed as follows:
    UserPayment=FinalPrice−minToken
    In this equation, if UserPayment≥0, the user needs to pay this amount, otherwise, the user will be refunded by userPayment.
  4. If UserPayment≥0, payment request will be sent to user, and $SC_{ACM}$ will receive the tokens from user’s wallet.
  5. $SC_{ACM}$ calculates the MNO and service provider’s shares from UserPayment as follows, and transfer tokens to each one.
    $$MNOshare=\left(UserPayment+minToken\right)\times Share_{MNO}^{PAYG}$$
    SPshare=(UserPayment+minToken)−MNOshare

## 4. Evaluation

### 4.1. Implementation

**Figure 9:**
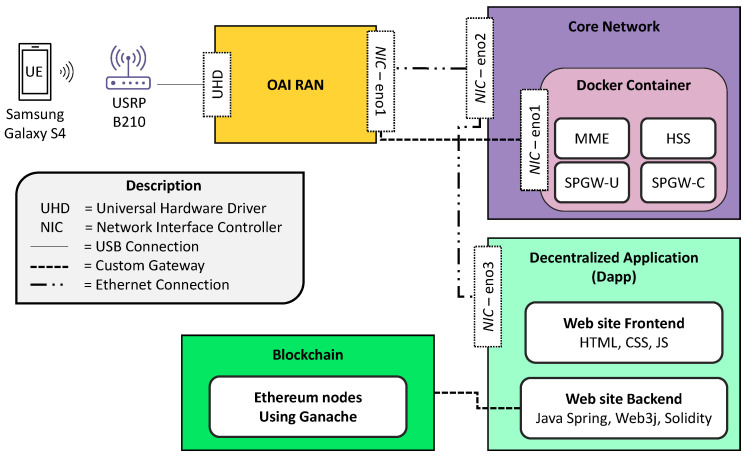
General architecture of our proposed system. The smartphone, USRP, OAI RAN, and Core network represent the cellular network testbed. The decentralized application and Blockchain represent the Blockchain technology implementation.

The overall implementation of our proposed system is depicted in Figure 9. This implementation can be discussed in terms of two sub-categories: Deployment of Cellular Network Testbed and Deployment of a Decentralized application on top of Blockchain.

#### 4.1.1. Deployment of Cellular Network Testbed

Our Github (https://github.com/nischalaryal/cellular-network-testbed-setup (accessed on 21 February 2023)) page provides a clear description for implementing the cellular network testbed. This testbed configuration requires three components: user equipment (UE), radio access network (RAN), and cellular network (CN) [26]. The primary goal of this configuration is to create an E2E setup, in which the UE equipment communicates with the CN via the RAN. The CN verifies the UE’s authenticity, and the UE verifies that it is connected to the correct network. Following successful authentication, the UE can connect to the internet via CN.

- **User Equipment:** We utilize the *Samsung Galaxy S4* smartphone with LTE network capability as our used equipment. A programmable SIM card connects the smartphone to the cellular network. The SIM card stores information such as secret keys, mobile country codes, mobile network codes, etc. This information helps in choosing the right network operator during the authentication process. We utilize a SIM card reader and pySim (https://github.com/osmocom/pysim (accessed on 21 February 2023)) software to program the SIM card and store the necessary information.
- **Radio Access Network:** We employ *OpenAirInterface5g* (https://gitlab.eurecom.fr/oai/openairinterface5g (accessed on 21 February 2023)) software to implement a softwerized RAN in COTS system. The COTS system configuration includes an Intel Core i7-6700 CPU running at 3.40 GHz, 16 GB of RAM, and Ubuntu 18.04 OS with a low latency kernel. The OAI RAN software includes the v1.1.0 git branch. We attach an *USRP B210* device to the system for radio communication between the UE and RAN. All the functionalities of the *USRP B210* device are handled by the USRP Hardware Driver (UHD), which is installed in the same system. In order to set up interfaces for communication with CN, OAI software provides numerous configuration files that contain data regarding PLMN and IP addresses. We utilize the configuration file named *enb.band7.tm1.50PRB.usrpb210.conf* to store the PLMN values, IP addresses, and network interfaces of RAN and CN.
- **Core Network:** We utilize *OpenAirInterface LTE*+ software to implement the core network in the COTS system. The COTS system configuration includes an Intel Xeon W-2102 CPU running at 2.90 GHz, 16 GB of RAM, and Ubuntu 20.04 OS. The OAI-based core network utilizes the master branch (https://github.com/OPENAIRINTERFACE/openair-epc-fed (accessed on 21 February 2023)) and the MagmaCore uses version 1.8 branch (https://github.com/magma/magma/tree/v1.8 (accessed on 21 February 2023)). All the modules of the core network are containerized using either Docker or VirtualBox. The MME functionality of CN initiates a connection with RAN and manages all requests arriving from UE via RAN. The information regarding the user, such as secret keys, is stored in the HSS database and is utilized during the authentication process.

#### 4.1.2. Deployment of the Decentralized Application via BCT

As shown in Figure 9, the decentralized application (DApp) part of the system implements a web service connected to BCT nodes. The web service consists of a front-end written in the Javascript language, connected to the back-end that is deployed using the Java Spring framework (https://spring.io/projects/spring-framework (accessed on 21 February 2023)). The connection to smart contracts and BCT nodes is handled by the web3j library (https://docs.web3j.io/ (accessed on 21 February 2023)). The smart contracts are deployed in Solidity language and compiled by the solc compiler (https://www.npmjs.com/package/solc (accessed on 21 February 2023)). The code of the deployed DApp is available online, in a GitHub repository (https://github.com/FaribaGhaffari91/AccessControlUsingBCT (accessed on 21 February 2023).

### 4.2. Performance Evaluation

To assess the implementation feasibility of the proposed method in private cellular networks, we designed a use case, in which the user uses their mobile connectivity (i.e., cellular network) to connect to the implemented DApp. To do so, we deploy a private cellular network environment to connect the user to the Data Network (DN). Note that the DN can be either the internet (i.e., when the DApp is deployed on the internet for public-use cases) or the MNO network (i.e., when the DApp is locally hosted in the MNO site for the private service-provisioning cases).

The performance analysis of the proposed method has been done in three connection and deployment types, as follows:

- Connecting the COST UE to the OAI-RAN and OAI-core networks, and DApp is hosted in MNO;
- Connecting the COST UE to OAI-RAN and magma core, and DApp is hosted in MNO;
- Connecting the COST UE to a public network, and DApp is hosted in either MNO or other third-party entities (available through the internet);

In the aforementioned testbeds, the following performance indicators are evaluated:

- The GAS usage: GAS is the fee that must be paid by the sender to submit transactions to the Ethereum network. GAS price in the public networks is defined in Gwei (i.e., as $10^{\left(-9\right)}ETH$, which is the real-time price of the Ethereum cryptocurrency). However, in the private or consortium Blockchains, this price can be modified by the actors and governors.
- The user-experienced latency ($T_{total}$): This time is the exact period it takes from sending the user’s request through the browser to getting the answer from the network.
- The transaction validation time (smart contract function execution time) ($T_{fn}$): this time defines the period that it takes for the network to execute all function(s) related to a specific request in the network and to return a transaction receipt for that. Note that $T_{fn}$ is dependent on the block time in the Blockchain network.
- The DApp latency ($T_{dapp}$): this time consists of the latency in the internal functions in DApp and the non-transaction calls to the Blockchain.
- The network latency ($T_{net}$): this time is the network latency of the user’s connection to RAN or core networks (i.e., $T_{net}=T_{total}-\left(T_{dapp}+T_{fn}\right)$).

These performance indicators are assessed in the following scenarios simulated in the system with COST users:

1. $Reg_{MNO}$: the registration of the MNO in the system including the deployment of $SC_{MNO}$;
2. $Reg_{SP}$: the registration of the service provider in the system, including the deployment of $SC_{SP}$ ad insertion of two services for each service provider, and the definition of two access policies for each service.
3. $Reg_{UE}$: the registration of the user in the system including the deployment of $SC_{U}$;
4. $Reg_{UE}^{Prepaid}$: the registration of the user in one of the available services with the prepaid pricing model.
5. $ACC_{UE}^{Prepaid}$: attribute-based access control of the user to access the registered prepaid service.
6. $Pay^{Prepaid}$: termination of the user’s access to the service and service provider’s payment to MNO on behalf of the user.
7. $Reg_{UE}^{PayG}$: the registration of the user in one of the available services with the PAYG pricing model.
8. $ACC_{UE}^{PayG}$: attribute-based access control of the user to access the registered PAYG service.
9. $Pay^{PayG}$: termination of the user’s access to the service, the user’s access time to the service, and their payment to the service provider and MNO accordingly.

**Figure 10:**
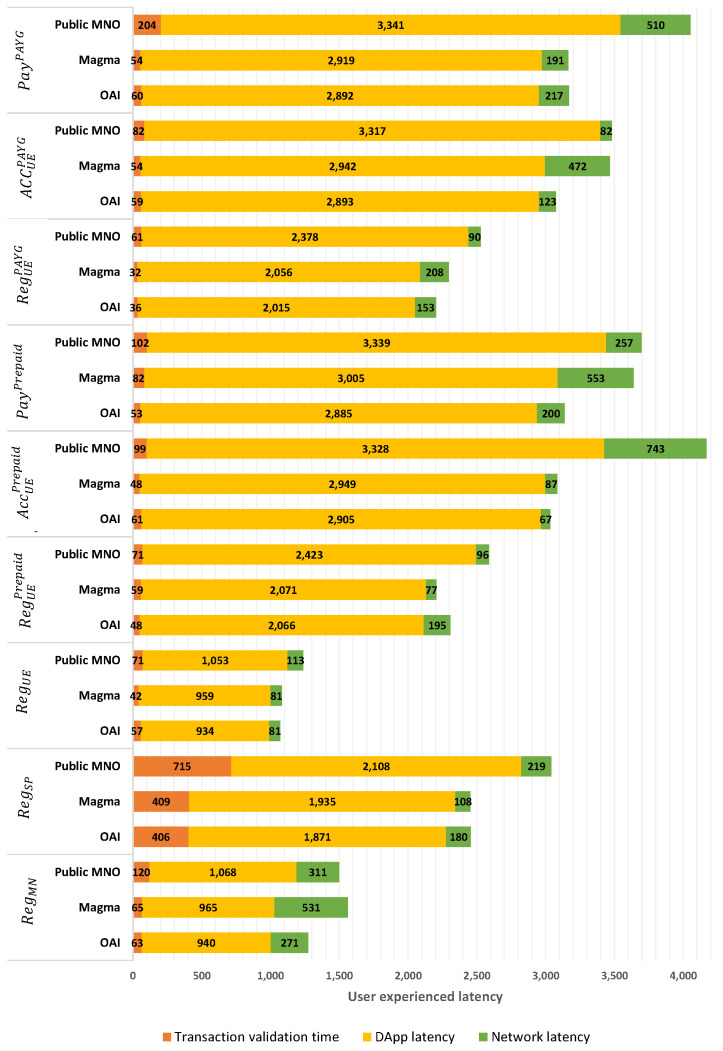
The user experienced latency of the system for the private or consortium DLT use-case, with low-security and high throughput requirements. Each bar in the figure represents the latency experienced by the user, and it is made up of network latency ($T_{net}$), DApp latency ($T_{dapp}$), and transaction validation latency ($T_{fn}$).

   User-experienced latency of the proposed method for private use cases is provided in Figure 10 regarding the different scenarios and the aforementioned performance indicators. Each bar in the figure indicates how the user experienced latency is decomposed to different times (i.e., $T_{net},T_{dapp},T_{fn}$).

Note that the utilized configuration for this analysis is applicable in private or semi-private use cases, where one/several companies govern the Blockchain and have the right to participate in consensus procedures and write them into the Blockchain. These companies offer services either exclusively to their specific customers (e.g., video streaming, remote meetings, storage, etc. for employees of one or several companies), or publicly for all users. Private-use cases here refer to a limited number of entities governing and managing the associated DLT, rather than a limited number of users. The security requirements in these use cases are higher due to the need to protect the system against intrinsic BCT attacks like a 51% attack [31]. Since participating nodes are already authenticated and known in the system, there is a minimum level of trust, making a simple consensus procedure sufficient for these networks. To simulate a simple consensus procedure, we set a minimum value for the block time, which defines the complexity of the consensus procedure. Figure 10 shows that the latency of function execution for user access to the system is very low compared to network and DApp latency. Additionally, the user’s experienced latency for access control is around 3 seconds, well comparable to existing centralized systems. In the real implementation of our method, many of the extra procedures in the DApp do not need to be executed, which can significantly decrease the value of $T_{total}$.

**Figure 11:**
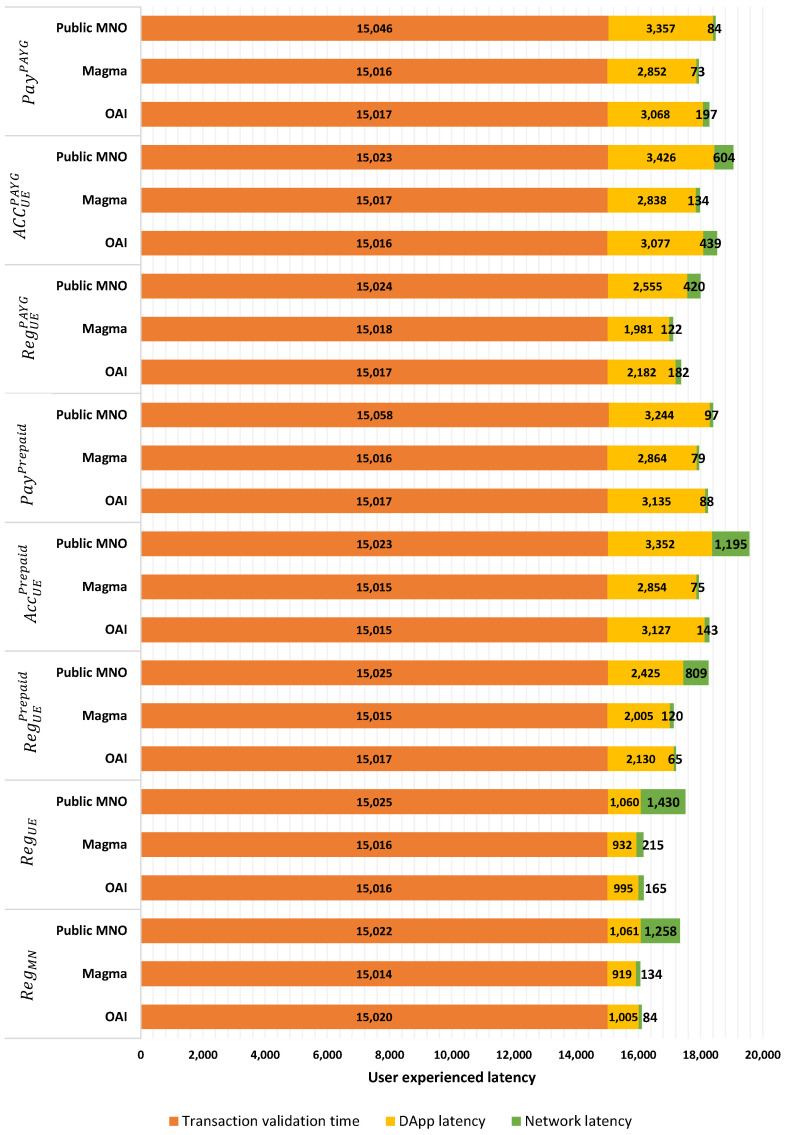
The latency of the system for public Blockchain-use case with high-security requirements. Each bar in the figure represents the latency experienced by the user, and it is made up of network latency ($T_{net}$), DApp latency ($T_{dapp}$), and transaction validation latency ($T_{fn}$).

Figure 11 provides the performance analysis for public-use cases where DLT governance is not in the hands of several organizations, and every micro-business or user can use it. In this scenario, no trust level can be assumed, and actors need to protect the system against BCT attacks [32] by enforcing more complex and secure consensus procedures. To simulate a complex consensus procedure, we choose a block time of 5 seconds, which is near to public networks. As shown in Figure 11, the latency of function execution to provide user access to the system is a significant portion of the user’s real experienced latency. To have a more secure network, we assume that nodes can update their ledger after two block times. Hence, when we select a block time of 5 seconds, the minimum time by which the user can see the result of her transaction is 15 seconds. This latency is higher than the expectation of the user and the experienced latency in centralized systems. A discussion on solutions to overcome this problem is provided in Section 5.2. In Figure 11, we avoided providing the latency data related to SP registration, as at least 15 transactions need to be validated and added to the blocks. Therefore, it is done at around 2.5 seconds, creating a significant difference from other data in the figure.

The GAS usage of the execution of smart contracts in each of the above-mentioned scenarios is provided in Table 1, based on the GAS used, and its price in Euro (€) at the time of writing the paper. As it is shown, the most expensive transactions will be sent to the network only once (i.e., $Reg_{MNO}$, $Reg_{SP}$, and $Reg_{UE}$). It is important to mention that in our use cases, since the system will not be deployed on top of public Ethereum, these prices are the maximum price that we can consider for the system. In consortium or private Blockchains, there is no need to define these prices for the transactions; they can be fully adjustable based on the application requirement.

## 5. Discussion

In this section, an essential discussion of different aspects of the deployment of the proposed method is provided. First, we propose some answers to potential questions concerning the real implementation of the system. Moreover, due to the ever-growing demands on the service provisioning domain, it is crucial to provide sufficient updatability and maintainability to the system, especially when we rely on an immutable system architecture, such as in the case of smart contracts. Furthermore, in the following subsection, we will also discuss two of the important points of discord in using BCT and smart contracts for access control procedures (i.e., the latency of the system and its storage complexity).

### 5.1. Real Implementation of the System

To deploy the proposed method in a real-world scenario for public or private cellular networks, several prerequisites need to be well-defined. For instance, (i) who are the governors or regulators of the ledger of our proposed blockchain design? (ii) Which entities are eligible to participate in securing such a ledger? (iii) How would the trust among actors (i.e., users, service providers, and MNOs) be addressed? and (iv) How can the maintainability and updatability of the smart contracts be guaranteed?

In terms of governance and security assurance of the system, the main actors in the proposed system are users, MNOs, and service providers. In our proposed method, the underlying ledger of our blockchain design can be implemented as a consortium DLT platform. Note that we propose to exclude users from participating in consensus and storing the entire ledger, as it would be challenging for users with power-constrained devices with limited and all-purpose storage to participate in the consensus of the DLT platform. Additionally, involving users in maintenance and securing operations not only requires an education phase (which can affect the user’s positive experience of using the system) but also raises higher security and privacy concerns. Therefore, we propose the following setting for method implementation in an operational scenario. The DLT itself should be governed by a consortium between MNOs and SPs. Smart contracts, such as $SC_{AB}$, which play a critical role in securing the connection of smart contracts (e.g., to prevent Reentrancy attacks), and provide a level of trustworthiness in the system, should be deployed once, with the approval of all entities, without the possibility of alteration in the next steps. Note that since $SC_{AB}$ acts as a distributed database, without any other operational role in the system, the agreement of a minimum number of participating actors would be sufficient in the first step of system setup.

Regarding the level of trust in the system, this concern can be broken down into the following sub-questions, with the provided proposals as follows:

- Trustworthiness in the immutability of prices advertised by the service provider: Since the advertised prices of the service providers are stored in $SC_{SP}$ with the service provider’s ownership, the first concern is how the other entities can be assured that this prices will not change without their agreement. To address this concern, we propose to implement a simple voting system for the entities that will be affected by the price changes. For instance, the following scenario is implemented in the current version: when an MNO registers in the service, its address is added to the list of beneficiaries in $SC_{SP}$. Updating the prices will be managed by updatePrice() function in $SC_{ReM}$. This function will execute a voting procedure, in which all the existing MNOs in beneficiaries list need to vote in a predefined time (the request will be sent to them as an event). Once the predefined tile is finished, or all beneficiaries voted, the decision will be based on the majority of the votes. Therefore, we can state that the prices are adaptable only in case of the agreement of all (or the majority of) the participants.
- Guaranteed payment to service providers and the MNOs after providing the service and network: Since, in this system, either the user will not pay the MNO (i.e., in prepaid services) or they pays based on their real utilization of the service (i.e., in PAYG pricing model), it is crucial for the MNOs, as well as service providers, to be sure about the payment. If the payment guarantee can not be provided in the proposed system, indeed, the service providers and MNOs will not be motivated to accept the new business model. To address this issue, we benefit from the trustworthiness of smart contracts through automatic execution in a deterministic way. In both prepaid and PAYG pricing models, we defined a fee that is blocked in the $SC_{ACM}$ at the time of the user’s request to access the service. The following approaches are proposed for two pricing models:
  – In the *prepaid scenario*, since the user already paid the service provider, this fee (i.e., $Share_{MNO}^{Prepaid}$) will be deducted from the service provider’s account and blocked in $SC_{ACM}$. After termination of the service usage, $SC_{ACM}$ will transfer the blocked money to MNO. It means the service provider is guaranteed to pay the MNO on behalf of the user.
  – In the *PAYG scenario*, since the user will pay both the MNO and service provider according to their usage, a minimum fee (i.e., minToken) will be deducted from the user’s account and blocked in $SC_{ACM}$. After termination of the service usage, $SC_{ACM}$ will transfer the blocked money to MNO and the service provider according to their share of this fee. In the current implementation of the system, if the user’s real usage is less than the blocked money, the user will be refunded, unless the remaining fee can be deducted from the user’s account. Other solutions are also possible to address this exceptional situation, provided as future directions.

Regarding the updatability of the system, there are two main concerns to consider. The first concern is related to the maintainability of smart contracts, which is a well-known challenge in the field. Once smart contracts are deployed, their code cannot be updated due to their immutability, which ensures their trustworthiness. To overcome this challenge, one solution proposed in [29] is to avoid hard-coding the addresses of smart contracts in the operational system of distributed applications. In our proposed method, we provide a solution that enables the update of contracts and replacement of the deployed smart contract with a new one. This can be done by defining $SC_{AB}$ as a reference smart contract to retrieve the current address of other smart contracts in the system instead of using hard-coded smart contract addresses. The admin(s) of the system can update this smart contract as needed.

The second concern is related to the flexibility of attributes and policies. Defining the access control policies inside one smart contract (e.g., $SC_{ACM}$ in our example) limits the flexibility of the system and increases the maintenance effort, as it restricts the access attributes to the pre-defined attributes at the time of smart contract deployment. To address this problem, we define the policies based on each service and design $SC_{PL}$ to keep the list of all policies mapped to their code. Each service has a mapping of its required access policies and attributes. At the time of access control, $SC_{ACM}$ fetches the related smart contract to the specific policy code, defined in the list of policies of the user’s requested service, and calls its verification function. Therefore, new policies can be defined in smart contracts, and their address will be recorded in $SC_{PL}$ for further access verification procedures.

### 5.2. Discussion on Latency and Storage Complexity

Low latency in access control procedures is a critical requirement in service provisioning use cases. In the use case of service provisioning for private purposes, where the participating nodes in the consensus procedure are authenticated and well-known for the system, the system latency can be significantly manageable by the level of complexity of the consensus procedure, as shown in Figure 10. Therefore, we skip discussing these scenarios. However, it is important to mention that due to our observations and the existing business model, this type of service provisioning may be more attractive to organizations and businesses.

Regarding the access control for service provisioning in public use cases, latency is one of the critical requirements. There are some proposals to address this issue as follows:

- Designing a tailor-made DLT for access control purposes can encourage the BCT community researchers to design a precise ledger with specific consensus models, block sizes, transaction fees, block times, incentives, and other specifications to make it possible to validate the higher number of transactions with high security in a given time. The main problem with this solution is that it needs lots of research and proof before application.
- Chain sharding is another novel solution that horizontally shards the chain to distribute the transaction loads among shards [33]. After the validation of transactions in the shards and generating the blocks, a smart contract would be utilized to merge the shard blocks to the main chain. This solution increases the throughput and decreases storage usage. Several sharding solutions are recently proposed that state the feasibility of this method. For instance, RapidChain [34] increased the throughput to 7380 TpS, in comparison with 15 to 20 in Ethereum, with 4000 participating nodes and 250 shards.

Regarding storage, BCT needs a considerable amount of storage in its full nodes to keep the ledger updated. Currently, the proposed method generates one to three transactions that are all stored in full-node storage. To address the storage complexity challenge of BCT, as mentioned earlier in the latency problem, the chain sharding solution can be applied. Chain sharding can be highly beneficial in case of storage requirements. In this technique, each shard functions independently of the other shards (i.e., it has its own block validation method, number of input transactions, and storage requirements). In each shard, the participating nodes are required to keep the transactions of their own shard. Moreover, optimizing the number of required transactions to be sent to the network is another proposal to address this challenge.

### 5.3. Discussion on Scalability

Scalability, security, and decentralization cannot be provided simultaneously due to the Blockchain trilemma. Scalability is the third characteristic that needs to be addressed due to the importance of all three factors in our use case and the knowledge that security and decentralization are provided in the proposed architecture (based on the intrinsic security features of smart contracts and the Blockchain). Access control solutions face a major obstacle because of the scalability issue with DLT on various systems. To increase throughput while expanding the number of concurrent transactions, scalability must be increased. Blockchain scalability can be divided into two categories: *horizontal* and *vertical*. Vertical scalability tries to expand the capabilities of participating nodes to achieve higher throughput, whereas horizontal scalability refers to the ability of Blockchain to raise the throughput (or at least not to decrease it) by adding new nodes. On the one hand, employing public DLTs might reduce system performance, raise costs, and add delays even though they are more horizontally scalable. Using permissioned DLTs appears to be a promising solution in light of these issues. On the other hand, the user’s identity and privacy may be in danger with permissioned DLTs. Therefore, a fascinating area for future work would be to propose a scalable customized DLT devoted to the use case that is a consortium of all participating providers. Although the aforementioned solution is beneficial in several aspects, it needs lots of research and proof before application. Another novel solution, which is also discussed in Section 5.2, is *sharding the chain* horizontally to distribute the transaction loads among shards [33]. This solution increases the throughput even by using the pre-examined and approved consensus models such as PoS, PBFT, etc. For instance, RapidChain [34] increased the throughput to 7380 TpS in comparison with 15–20 in Ethereum with 4000 participating nodes and 250 shards.

## 6. Related Work

Many recent solutions have been proposed on the topic of efficient access control for service provisioning. However, a considerable portion of the state-of-the-art research in access control is dedicated to centralized systems, which do not have a direct link to service provisioning in cellular networks. While centralized solutions are easier to implement and offer low latency and storage efficiency, they suffer from having a single point of failure, low scalability, low availability, and low non-repudiation. [35,36].

There are several decentralized access control solutions in the related literature. Shafeeq et al. [37] proposed an ABAC mechanism that uses Tangle [38] to store policies and access attributes in a DAG-based DLT solution. In their method, owners define and manage the access rules, security policies, and authorization granularity over their assets and store them in the DLT. Upon receiving an access request, the owner sends the authorization token to the requester only if the requester meets the conditions defined in the access control policy. Zhang et al. [39] proposed a hierarchical model for sharing and accessing healthcare data. In this method, BCT is used as a distributed ledger of permissioned clients to store verified codes of ciphertexts and record the hash values of auditing logs. Qin et al. [40] proposed an ABAC method to share data in the cloud environment by storing the access policies in the BCT. In this system, a certification authority (CA) manages the security of the entire system. First, the CA issues an attribute key to the user and the system in the smart contract, which has an expiration time. Then, in the access control phase, the data owner uploads the ciphered text to the system, and the system invokes the contract to obtain the user’s valid attribute set. BlendCAC [35] is another system that uses smart contracts for storing the access control matrix of the CapBAC model. Each node interacts with the smart contract through the provided contract address and the use of remote procedure calls.

Rather than using BCT as a distributed database, the following works exploit this technology as an access validation and enforcement solution. In the field of cellular networks and telecommunications, Ling et al. [41] proposed B-RAN, an ABAC model implemented as a BCT for access management in Radio Access Networks (RAN). This method implements self-organized access for users and providers, along with enabling mobility management. The user and network provider reach an agreement on cost and digitized spectrum assets, written in a smart contract. After validating the smart contract concerning these parameters, the user can use the resource for a limited time, and the access point will automatically receive payment for the access. Moreover, Ling et al. [42] proposed a BCT-based medium access control method. Suk-hodolskiy et al. [43] presented a system that manages user access via smart contracts, containing the location of the object, access policy, and additional owner’s information. One obstacle in the adoption of this method is the incompatibility between the immutability of typical BCT designs and attribute updates or revocations, which is addressed in [44]. Wang et al. [45] proposed a fine-grained access control for cloud storage. In this method, the owner deploys a smart contract to store the essential data of the file. To grant access, the owner defines the expiration time and a secret key and adds them to the smart contract. Then, the owner sends the data to the user, who can download and decrypt the file.

The previous work by Ghaffari et al. [46] proposes an access control method for service provisioning based on BCT. In this method, a single network provider supplies a BCT-based system to provide an access control solution as a service for other service providers. This approach removes the single point of failure in the access control procedure, improves scalability, immutability, integrity, and provides a new business model for service provisioning. However, this early version of the work suffers from several issues such as storage complexity due to the non-linear deployment of smart contracts over system entities, low maintenance capability regarding policy updates, revocation complexity, and a limited number of network providers.

This paper presents a solution to the aforementioned issues. The proposed solution decreases the order of smart contracts deployment to linear complexity. It maps registered services and their information explicitly into a single smart contract for each user, service provider, and mobile network operator. Instead of hard-coded policies in the access manager smart contract, the proposed solution deploys one smart contract for each policy. Each service has a list of policies that can be applied while validating the user’s access to the system. The user revocation procedure is straightforward since specific smart contracts are now explicitly mapped for each user. Additionally, the retrieving complexity of users’ registrations for each specific service is constant, which supports an unlimited number of network providers to participate in the system. The new solution also extends payment methods to prepaid and PAYG schemes, providing new business models for service providers’ registration in the system and decreasing the cost of the smart contract’s function execution.

## 7. Conclusions

There is growing interest in widening blockchain technology (BCT) towards new applications. BCT assures decentralized security and trust. We have reviewed some of the main technological aspects of BCT and presented a concrete application. We have proposed a decentralized access control-as-a-service solution for private cellular networks. The solution builds a distributed access control system that can be used by service and content providers. We have shown how our approach can provide new business models. The design and implementation of our method in a real-world scenario have been presented and evaluated. Discussion and comparison to related work have also been addressed.

For future work, we plan to extend our implementation in a number of ways. First, we plan to improve the assessment and analysis of the proposed method, including a more detailed study of the cost and performance of the system. Likewise, we plan to include some additional scenarios and platforms (e.g., Hyperledger Fabric and Quorum), as well as additional consensus models, to balance the parameters of the system. Other ideas include extending to a hybrid pricing model and proposing a new BCT-based pricing model. For instance, instead of the user’s direct payment by money, the user’s payment to the MNO or SP can be done by paying the transaction fee while sending a transaction to the system. The new business model can be defined to set a price for sending transactions to the system with the price of the service.

## Figures and Tables

**Table 1:** Environment specifications.

Parameter	GAS Used	Price in €
$Reg_{MNO}$	320,065	0.47
$Reg_{SP}$	463,673	0.69
Add new service by SP	135,841	0.20
Add new policy for service	51,529	0.07
$Reg_{UE}$	343,685	0.51
$Reg_{UE}^{Prepaid}$	182,172	0.27
$ACC_{UE}^{Prepaid}$	95,671	0.14
$Pay^{Prepaid}$	68,307	0.10
$Reg_{UE}^{PayG}$	48,331	0.07
$ACC_{UE}^{PayG}$	95,787	0.14
$Pay^{PayG}$	75,831	0.11

## Data Availability

Not applicable.
